# Editorial Expression of Concern: Baicalein protects HT22 murine hippocampal neuronal cells against endoplasmic reticulum stress-induced apoptosis through inhibition of reactive oxygen species production and CHOP induction

**DOI:** 10.1038/s12276-021-00598-8

**Published:** 2021-04-16

**Authors:** Dae-Myung Jue

**Affiliations:** Biochemistry, Catholic University of Korea, Seoul, Republic of Korea

The Editors are issuing this Editorial Expression of Concern to alert readers that concerns have been raised regarding multiple duplications in Figs. 2B, 3A–D, 4B, and 5C, D in this article^1^.

The authors have provided documentation concerning the outcome of an investigation by The Kyung Hee University Inquire Committee that found no problems in any of the figures. The Editorial Office has been unable to confirm such findings directly with authorities at the institution in accordance with standard practice. Given the nature of the concerns raised, the Editor-in-Chief wishes to alert the readers to interpret the results presented here with caution.

Corresponding authors Insug Kang and Eui-Ju Yeo stated on behalf of all co-authors that they agree to this Expression of Concern.

## References

[1] Choi JH (2010). Baicalein protects HT22 murine hippocampal neuronal cells against endoplasmic reticulum stress-induced apoptosis through inhibition of reactive oxygen species production and CHOP induction. Exp. Mol. Med..

